# Homœopathy

**Published:** 1854-02

**Authors:** 


### HOMŒOPATHY.

It is a question with many persons whether it is good policy in our profession to notice the medical delusions that are continually springing up; whether a consequence greater than they would otherwise attain is not given to them by the opposition of scientific physicians. There are those who would leave them, as the traveller does the noisy insects in his path, to die a natural death. They succeed each other rapidly, to flourish for a season, and then pass into the limbo of forgotten things. But another party would pursue a different course. They insist upon the expediency of exposing each new humbug as it is blown into existence; and for ourselves, we confess, we incline, not exactly to the belligerent system, but to giving the people light. If we as a profession withhold it, from what quarter can it come? Unless the false systems ever and anon arising are exposed by medical men, how are they to be exploded?

This view of the matter is taken by Prof. Simpson, who, deeming a further and fuller exposure of Homœopathy expedient, has written the most elaborate refutation that the popular delusion has yet called forth. The following capital anecdote is given, and illustrates the manner in which Homœopathists are occasionally made. The professional friend alluded to by Dr. S., is Prof. Henderson, of Edinburg, one of the most noted Homœopathists in Great Britain.

"Some eight or ten years ago, and old schoolmate of Dr. Simpson having begun business as a homœopathic druggist in Liverpool, kindly sent Dr. S. a present of a small box of homœopathic medicines, and a very beautiful painted box it was. During the time it was in Dr. S's. possession, he put it only to one use, viz., he gave it as an occasional plaything to his eldest son, who was then a child. The boy, revelling in his permitted amount of mischief, used in his sport to uncork the small bottles, empty their globules into a heap, and then refill the bottles from the general mass. Of course, this had speedily the effect of altering and disarranging the contents of the entire lilliputian drug shop; the globules pertaining to the different bottles were more or less thoroughly mixed together; and sometimes, when the child was tired of his occupation, others at last refilled the bottles from the general heap. A professional brother happening to call at Dr. S's. house one day when Dr. S. was absent from home, saw the box, and put it in his pocket. Many weeks afterwards, the new proprietor of the box met Dr. S., and told him that he had been trying to practice homœopathically, at which Dr. S. expressed his regret; and he added that he had seen some wonderful effects and cures from using the drugs contained in Dr. S's own former homœopathic box! Wrongly, perhaps, as Dr. S. now thinks, he did not at the time tell this physician that the globules of the bottles which he had been using were elaborately commixed; but the whole struck him as so good a joke at the moment, that he thought he would reserve it to bring it out upon his friend on some future and more ripe occasion, for the purpose of laughing him out of his homœpathic delusion. But, unfortunately, matters hastened rapidly on; the physician became more and more a homœopathist; and then it became too serious a matter to joke about, when he actually published a list of supposed homœopathic cures."

